# One-Year Follow-Up of an Inframalleolar Collateral Artery Bypass

**DOI:** 10.7759/cureus.36192

**Published:** 2023-03-15

**Authors:** Sunil Rajendran, Harishankar Ramachandran Nair

**Affiliations:** 1 Department of Vascular Surgery, Starcare Hospital, Kozhikode, IND; 2 Department of Vascular Surgery, Malabar Institute of Medical Sciences, Kozhikode, IND; 3 Department of Vascular Surgery, King’s College Hospital, London, GBR

**Keywords:** inframalleolar bypass, chronic critical limb ischemia, pedal bypass, collateral bypass, ankle collateral

## Abstract

Successful revascularization and restoration of blood flow to one of the pedal arteries is the mainstay to prevent major limb amputation. Here, we report a rare case of successful bypass to the inframalleolar ankle collateral artery in a middle-aged female with rheumatoid arthritis presenting with gangrene of the toes of the left foot. A computed tomography angiography (CTA) demonstrated normal infrarenal aorta, common iliac, external iliac, and common femoral arteries on the left side. The left superficial femoral, popliteal, tibial, and peroneal arteries were occluded. Extensive collateralization was noted in the left thigh and leg, with distal reformation in large ankle collateral. A successful bypass was done using from the common femoral artery to the ankle collateral using the great saphenous vein harvested from the same limb. At a one-year follow-up, the patient was symptom-free, and a CTA showed a patent bypass graft.

## Introduction

Chronic limb-threatening ischemia (CLTI) is an extreme spectrum of peripheral occlusive vascular disease and is often associated with mortality, amputation, and impaired quality of life [1,2]. CLTI is often an end result of multilevel occlusive vascular disease involving multiple infra-inguinal arteries [1]. Successful revascularization and restoration of blood flow to one of the pedal arteries is the mainstay to prevent major limb amputation [3]. Here, we report a rare case of successful bypass to the inframalleolar ankle collateral artery.

## Case presentation

**Figure 1:**
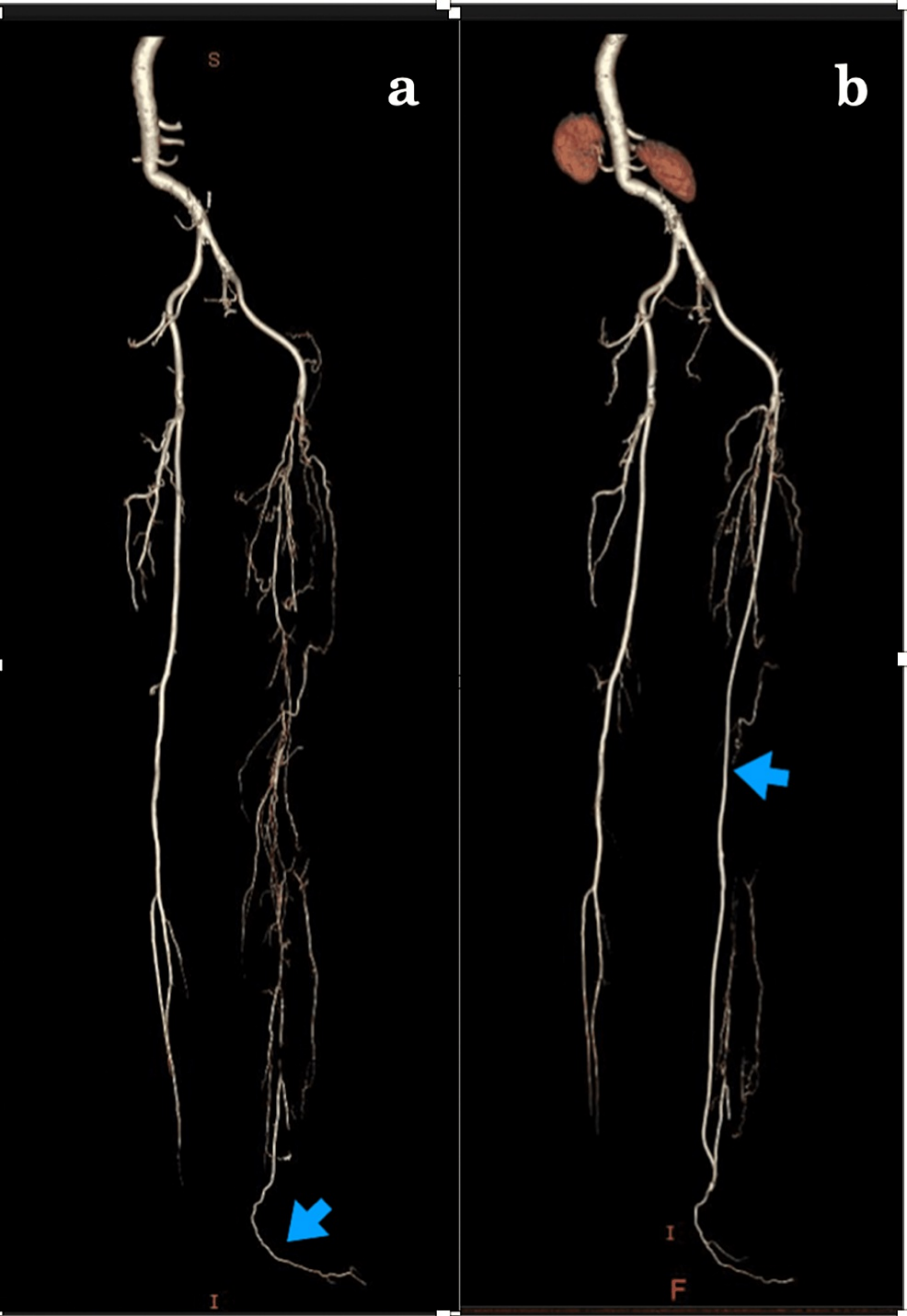
(a) Computed tomography angiography showing a long-segment occlusion of superficial femoral, popliteal, and tibial arteries, with distal reformation in a large ankle collateral (arrow). (b) Computed tomography angiography at the one-year follow-up showing patent vein bypass (arrow).

A 73-year-old woman presented with gangrene of the left little toe and an ischemic ulcer in the third and fourth webspace. She was on chronic treatment for rheumatoid arthritis but did not have any other comorbidities. A computed tomography angiography (CTA) demonstrated normal infrarenal aorta, common iliac, external iliac, and common femoral arteries on the left side. The left superficial femoral, popliteal, tibial, and peroneal arteries were occluded. Extensive collateralization was noted in the left thigh and leg, with distal reformation in large ankle collateral (Figure 1a). The arterial tree of the right lower limb was grossly normal. As an endovascular option was not deemed possible, she was planned for a femoral to inframalleolar bypass to the collateral artery. Under regional anesthesia, the ankle collateral was approached through a curved incision just posterior to the medial malleolus. The distal posterior tibial artery was occluded, and a good-sized distal target artery was located between the Achilles tendon and inferior to the medial malleolus. The common femoral artery (CFA) was exposed through a standard vertical groin exposure. The great saphenous vein was harvested from the same limb and reversed to prepare a long conduit. The CFA was clamped after systemic heparinization with 5,000 units of intravenous heparin. A vertical arteriotomy was made in the CFA, and proximal anastomosis of the vein graft was done with a 7-0 polypropylene suture. After completing the proximal anastomosis, the vein graft was tunneled sub-sartorially to behind the knee joint and then to the posterior compartment of the leg, and finally to the ankle. The ankle collateral artery was clamped with fine bulldog clamps, and after making a 5 mm long arteriotomy, the distal anastomosis was done using a 7-0 polypropylene suture. Wound debridement and amputation of the fifth toe were done two days after the revascularization procedure. While in the hospital, 5,000 units of heparin three times daily were administered, and at the time of discharge, she was prescribed 150 mg of aspirin once daily and 2.5 mg of rivaroxaban twice daily. At the three-month follow-up, her wound in the foot was healed completely. At the one-year follow-up, she was symptom-free, and a CTA showed a patent bypass graft (Figure 1b).

## Discussion

Critical limb ischemia is the most severe pattern of peripheral artery disease and is associated with a high risk of major amputation, cardiovascular events, and death [4]. With the increased incidence and prevalence of diabetic and aging populations, there has been a significant increase in the number of infra-popliteal artery occlusive diseases [5]. When CLTI involves the infra-popliteal arteries, there is often relative sparing of peroneal and dorsalis pedis arteries, which have been successfully used as targets for revascularization [1]. However, in extreme circumstances, bypasses are also done to inframalleolar arteries such as plantar and tarsal arteries [6]. Although inframalleolar bypass is technically challenging, acceptable limb salvage rates have been reported [3].^ ^The usual target arteries for such bypasses are dorsalis pedis, or even plantar arteries [3]. In extreme situations, bypasses are also done to the named collateral arteries such as descending genicular artery or median sural artery [7]. In the absence of the above-mentioned target arteries, there has been an isolated case report of bypass to an unnamed collateral artery around the knee [8]. However, bypasses to inframalleolar collateral arteries are hitherto unreported.

Patterns of compensating anastomosis around the ankle have been described in patients with CLTI [9]. Our patient probably had a hypoplastic posterior tibial artery with hypertrophy of the distal peroneal artery collateral continuing as the plantar artery, as described by Zwass and Abdelwahab [10]. In this report, we have described a successful bypass grafting to the above-mentioned collateral artery. The absence of a patent posterior tibial artery at the ankle intraoperatively supported our conviction that the artery in question was a collateral branch.

## Conclusions

Critical limb ischemia is the most advanced form of peripheral arterial disease, which demands prompt revascularization to prevent major limb amputation. Predominant involvement of infra-popliteal arteries poses great challenges in successful revascularization, which is often successfully managed by endovascular techniques. However, in the face of failed endovascular procedures and in the absence of any suitably named arteries, revascularization to an appropriate collateral artery appears to be a durable procedure.
